# Assessing health system responsiveness to the needs of older people

**DOI:** 10.26633/RPSP.2021.127

**Published:** 2021-09-30

**Authors:** Emmanuel Gonzalez-Bautista, Patricia Morsch, Mallika Mathur, Ângelo José Gonçalves Bós, Carolina Hommes, Enrique Vega

**Affiliations:** 1 Pan American Health Organization/World Health Organization Washington D.C. United States of America Pan American Health Organization/World Health Organization, Washington, D.C., United States of America; 2 Pontifical Catholic University of Rio Grande do Sul Porto Alegre Brazil Pontifical Catholic University of Rio Grande do Sul, Porto Alegre, Brazil

**Keywords:** Health information systems, health systems, indicators of health services, aged, Barbados, Brazil, Chile, Mexico, Sistemas de información en salud, sistemas de salud, indicadores de servicios, anciano, Barbados, Brasil, Chile, México, Sistemas de informação em saúde, sistemas de saúde, indicadores de serviços, idoso, Barbados, Brasil, Chile, México

## Abstract

**Objective.:**

To identify key indicators that will allow empirical measurement of a health system’s responsiveness to older people.

**Methods.:**

We conducted a series of consultations with experts to develop a relevant list of indicators. Concept mapping was used to devise the list, including the steps of preparation, brainstorming and structuring. Additionally, four countries were used as national case studies to test the feasibility of measuring health system responsiveness with readily available national-level data (Barbados, Brazil, Chile, and Mexico).

**Results.:**

Our study resulted in a list of 25 indicators scored with high usefulness for informing public policy, 10 of which were also categorized as being of high availability. National case studies were useful to assess the feasibility of measuring health system responsiveness in different settings.

**Conclusions.:**

Responsiveness can be comprehensively assessed by (i) approaching the intrinsic features of the system via its inputs, outputs, and outcomes, and (ii) measuring the impact of the system on meeting the needs of older people in terms of their health, financial protection, and expectations. Further consensus is needed to develop a list of core indicators that could be used as a baseline for measuring a health system’s responsiveness to the needs of older people.

## RESUMEN

**Objetivo.:**

Determinar qué indicadores clave permitirán la medición empírica de la capacidad de respuesta de un sistema de salud a las personas mayores.

**Métodos.:**

Se llevó a cabo una serie de consultas con expertos para elaborar una lista de indicadores pertinentes. Para elaborar la lista se usó un mapeo conceptual, que incluyó los pasos de preparación, tormenta de ideas y estructuración. Además, se emplearon cuatro países en estudios de casos nacionales para evaluar la viabilidad de medir la capacidad de respuesta del sistema de salud con datos fácilmente disponibles a nivel de país (Barbados, Brasil, Chile y México).

**Resultados.:**

Se obtuvo una lista de 25 indicadores que se clasificaron como de alta utilidad para la fundamentación de políticas públicas, diez de los cuales también se categorizaron como de alta disponibilidad. Los estudios de casos nacionales fueron útiles para evaluar la viabilidad de medir la capacidad de respuesta del sistema de salud en diferentes entornos.

**Conclusiones.:**

La capacidad de respuesta se puede evaluar de manera integral *a*) abordando las características intrínsecas del sistema mediante sus insumos, resultados inmediatos y resultados intermedios, y *b*) determinando el efecto del sistema en la satisfacción de las necesidades de las personas mayores en cuanto a su salud, protección financiera y expectativas. Es necesario un mayor consenso para elaborar una lista de indicadores centrales que puedan usarse como línea de base para medir la capacidad de respuesta de un sistema de salud ante las necesidades de las personas mayores.

## RESUMO

**Objetivo.:**

Identificar os principais indicadores que permitem medir de forma empírica a resposta de um sistema de saúde às pessoas idosas.

**Métodos.:**

Realizamos uma série de consultas com especialistas para desenvolver uma lista relevante de indicadores. O mapeamento de conceitos foi utilizado para criar a lista, incluindo as etapas de preparação, discussão de ideias e estruturação. Além disso, quatro países foram usados como estudos de casos nacionais para verificar a viabilidade de medir a capacidade de resposta do sistema de saúde com dados de nível nacional prontamente disponíveis (Barbados, Brasil, Chile e México).

**Resultados.:**

Nosso estudo resultou em uma lista de 25 indicadores pontuados como de grande utilidade para a informação para políticas públicas, 10 dos quais foram também classificados como de alta disponibilidade. Os estudos de casos nacionais foram úteis para avaliar a viabilidade de medir a capacidade de resposta do sistema de saúde em diferentes meios.

**Conclusões.:**

A capacidade de resposta pode ser avaliada integralmente por meio de (i) abordagem das características intrínsecas do sistema por meio de seus insumos, resultados e desfechos, e (ii) medição do impacto do sistema na resposta às necessidades das pessoas idosas em termos de saúde, proteção financeira e expectativas. É necessário maior consenso para desenvolver uma lista de indicadores básicos que possam ser usados como linha de base para medir a capacidade de resposta de um sistema de saúde às necessidades das pessoas idosas.

The Region of the Americas is one of the fastest aging regions in the world, with increasing demands for acute and long-term care (1). Demographic and epidemiological transitions across the Region are highlighting the different levels of preparedness to address the needs of an aging society, such as efforts to maintain older people’s functional ability, and to manage chronic conditions and infectious disease outbreaks (2). In the context of a highly heterogeneous older population, the current performance of health systems has led to gaps between the needs of older people and their utilization of health care services (3). Bridging that gap requires health systems to become more dynamic, malleable, and people centered to foster integrated care that can better respond to the needs of the older population (4). Since the approval of the Strategy for Universal Access to Health and Universal Health Coverage in 2014, the Pan American Health Organization (PAHO) has been searching for strategic interventions to guide the design and implementation of policies and methods for strengthening or transforming health systems and achieving equitable access to health (5).

Population aging, as one of the greatest challenges in public health (6), requires a transformation in the way in which health systems are organized and funded to ensure affordable access to services that focus on the needs and rights of older people (7), such as making general adaptations to service provision, ensuring the health system’s ability to meet basic needs, and taking into consideration the personal goals of older adults when setting treatment goals.

Specific frameworks provide guidance for health services to help them adapt to population aging, for example, the building blocks approach (8), the responsiveness framework (4), the fit for aging approach (9), the 4M framework for delivering high-quality care to older people (10) and the integrated care for older people approach (11). Also, PAHO has developed a universal health framework (UHF) (12) that includes elements crucial to the transformation of health systems, such as (i) human resources (i.e. health professionals); and efforts to (ii) ensure full coverage and maximum utilization; (iii) improve financing, taking into account equity and efficiency (i.e. financing for the system and financial protection for the users); and (iv) strengthen multisectoral coordination (e.g. for long-term care). Regardless of the conceptual developments provided by these frameworks, a structured methodology that comprehensively assesses the level of responsiveness of health systems to the needs of older people is lacking.

According to a review by Khan and colleagues, responsiveness includes the feedback loops between the users and the health system, and also social accountability and community accountability in which responsiveness to the needs of older adults is a result of the interaction of broader governance and health system contexts (13). Similarly, the model proposed by Mirzoev and Kane (4) and the UHF (12) integrate the contextual factors and the determinants of the health system’s response, and how both elements interact with the expectations of individuals, families and the community.

Regardless of the conceptual developments around health system responsiveness, recent reviews have reported the following gaps: (i) few empirical studies have attempted to measure responsiveness, most of them focusing instead on users’ self-assessments of their satisfaction, and none specifically targeting the needs of older people (4); (ii) there is a need to develop more complex indicators to measure responsiveness; and (iii) there is also a need to empirically test the suitability of existing frameworks to assess specific health system priorities (13).

Our project seeks to address these empirical evidence gaps and integrate a 360 ° view of responsiveness using indicators of health systems and of older people’s needs and expectations. Our objective is to identify the key indicators that will allow for empirical measurement of health system responsiveness to address the gaps.

## METHODS

Drawing on concept mapping methodology (14), we conducted a series of consultations with experts in health systems and older people’s care to develop a list of indicators necessary to measure a health system’s responsiveness to older people. The concept mapping steps used were preparation, brainstorming, and structuring. Concept mapping has been proposed for use as a structured way to develop a framework for planning and evaluation (14). It has been used in the field of health systems to develop an integrative framework for delivering people-centered health care services (15).

The invited experts provided updated knowledge about monitoring and measuring health systems and the needs of older people. Meetings also involved experts from complementary disciplines, such as health systems management, to develop a comprehensive view of the measuring and monitoring processes.

### First expert meeting: August 20, 2017

The specific aim of the meeting was to generate an extensive list of indicators of health system responsiveness that were based on the UHF (12) and adapted to the needs of older people.

#### Preparation.

Fifteen participants invited to the expert meetings were selected by the Healthy Aging Unit of PAHO’s regional office based on their experience in geriatrics (as determined from their clinical or research profiles), health services and systems (i.e. managers of national hospitals, agents from national health authorities working in the area of aging and life course, and technical officers working in monitoring and evaluation), and in multilateral health organizations (i.e. PAHO staff and consultants). Further selection criteria included the absence of conflicts of interest and availability to attend. The selection of experts was reviewed and validated by PAHO’s office in Washington, D.C., and the PAHO office in the expert’s country of residence in cases in which they were government employees. The experts were appointed on an individual basis if they were not working as public employees, and these experts were mainly academics. Invitations to public employees were sent to national health authorities via a PAHO/WHO Representative with a description of the profile needed.

The meeting began with an overview of aging in the Americas and how it is linked to health systems. After this overview, the UHF was presented by staff from PAHO’s Health Systems and Services Unit; this included an explanation of the framework’s structure and a review of the indicators proposed, which are not focused on the needs of older people. Finally, a question and answer session occurred (12).

#### Brainstorming.

Facilitators (specialists at PAHO) conducted a brainstorming session to generate a list of indicators to measure the dimensions of the UHF from the point of view of older people and their needs, and the effects of population aging. Participants were encouraged not to make judgements about the correctness of suggested indicators. Facilitators captured the indicators in a computer document and projected them for all to see. The session concluded when participants agreed that the indicators were sufficient to capture all of the dimensions of the framework.

#### Structuring.

Although a quantitative structuring process was not carried out, the final list of indicators was sorted according to the dimensions of the UHF. Indicators were generated, moving from impact to outcomes, outputs, inputs and finally to the macro-determinants that go beyond the health system. Once the list of indicators was integrated, participants individually revised and approved the list.

### Second expert meeting: November 29, 2017

The specific aim of this meeting was to identify high-priority indicators of the responsiveness of health systems to the needs of older people, according to their relevance and usefulness for informing policy and their availability in national health system monitoring platforms.

#### Preparation.

The same procedures were used to select participants for this meeting as for the first meeting. Most of the participants in the first meeting were also available for the second. The organizers decided to replace missing participants with other invitees who met the criteria for inclusion.

Before the meeting, the list of indicators was shared with the participants and they were asked to check if the indicators were available within their national data repositories.

#### Rating.

Participants completed individual surveys to rate the indicators based on their usefulness for informing policy and their availability (Likert scale-type score, ranging from 0 to 5 for each criterion). The organizers computed and presented the results, and this was followed by a discussion session focused on those indicators with the highest and lowest scores. Participants were able to reconsider any of their scores based on the results of the discussion.

#### Structuring.

Two clusters of indicators were of most interest: (i) those with high relevance and high availability – that is, theoretically ready to be monitored; and (ii) those with high relevance and low availability – that is, important indicators worthy of being advocated for collection and analysis.

### National case studies

The specific aim of developing the national case studies was to test the feasibility of measuring health system responsiveness with readily available national-level data.

Countries were selected for the case studies if they had a population profile in the advanced phases of the demographic transition (i.e. among the largest percentage or absolute number of people aged 65 years and older) (16) and had requested technical cooperation from PAHO to address the impact of population aging on their health system.

Based on the indicator list obtained from the second meeting, we searched for individual-, subnational- and national-level data from four countries between December 2017 and May 2020 (Barbados, Brazil, Chile, and Mexico). Our team performed web-based searches using traditional search engines to detect grey literature, peer-reviewed articles and publicly available data sets. In parallel, a technical officer in health system monitoring from national counterpart offices searched for readily available data to compute either the numerator and denominator, or for the already estimated national level indicator.

## RESULTS

### First expert meeting

A comprehensive list of 62 indicators was developed, and the full list is available from the authors upon request. In total, there were 7 indicators of impact and 34 of intermediate outcomes, including 5 measuring coverage, 2 for access to preventive services, 8 for access to treatment services, 10 measuring quality and person-centered services, 3 representing human resources in health, and 6 measuring financing and financial protection. Additionally, there were 10 indicators of social determinants of health as contextual factors and 11 miscellaneous indicators.

Our team learned that less than half of the suggested indicators were ready to operationalize for data collection in any given health system. Approximately 35% (22/62) of the indicators were not defined specifically enough, and 20% (12/62) of them were aspirational, in the sense that it would be ideal to measure in them in the long term. Furthermore, we developed technical data sheets for the indicators, with their definitions, rationale, and numerators and denominators (available upon request). We concluded that the indicator list needed to be further reviewed to prioritize the most relevant indicators and avoid overburdening health systems with data collection and analysis.

### Second expert meeting

Nine out of fifteen participants responded to the survey. From the initial list, 25 indicators were scored as having high usefulness for informing public policy. Ten of these were also categorized as having high availability, and two were found to have high usefulness but very low availability, and thus these were proposed as targets for increasing monitoring capacity within national health systems (Table 1).

During the discussions, participants agreed that the number of core indicators in a responsiveness tool should be low (ideally less than 10) to increase the applicability of the systematic measurement of responsiveness at the national level.

### National case studies

The aim of the national case studies was not to compare the countries, but rather to present an overview of the availability of indicators in different scenarios. The selected countries for the case studies were Barbados, Brazil, Chile, and Mexico, based on the criteria in the Methods section. In Brazil, Chile, and Mexico, there were at least two nationally representative surveys related to health and aging, as opposed to Barbados, where there was only one study. The results of the case studies were organized in line with the UHF (12).

In 2018, in Chile, there was 1 geriatrician for every 48 000 older people, but 1 in 3 frail older people aged 80 years or older had not received a geriatric consultation. Also in 2018, in Brazil, there was 1 geriatrician for every 15 400 older people. In Mexico in 2018, there was 1 geriatrician for every 25 000 older people. In Barbados approximately 14% (1/7) of academic programs explicitly included geriatrics or gerontology in their curricula, while in Brazil only 41% (74/180) of medical schools offered training in the field of geriatrics.

In terms of health system coverage and utilization, Barbados, Brazil and Mexico reported more than 90% coverage of the influenza vaccine, while in Chile the coverage decreased from 98% in 2010 to 54% in 2016. Also, functional evaluations of older people had not been systematically implemented in any of the countries at the time the data were collected. In Brazil one in three older people presented with some deterioration of function, but only 0.3% of the older population had received care based on the Caderneta de saúde da pessoa idosa (a framework for comprehensive assessment and follow up in primary care). In 2011, 23.7% of older Brazilians reported polypharmacy, and in Barbados the average number of medicines per prescription was three; neither country had a published strategy to avoid inappropriate polypharmacy.

In terms of improving financing to ensure equity and efficiency, in Barbados out-of-pocket health care expenses were two times higher in households with one or more older people than in households without any older adult. In Brazil, 15% of the total income of families with at least one older person was spent on health care. In Chile, although out-of-pocket spending on health decreased from 42.5% to 32.2% from 2005 to 2015, households in which there was at least one older adult experienced catastrophic expenses (i.e. spent more than 30% of their income) three times more often than households that did not include an older adult. In Mexico, 41% of older people were living in poverty in 2016, and 19% could not write or read, which is comparable to data from Brazil. In both countries the proportion of older people reporting feeling unsafe while outdoors was around 35%.

**TABLE 1.** Indicators of health system responsiveness identified by expert consultants as having high priority because of their usefulness to inform public policy and their availability in national information systems

**Indicators with high usefulness**	**Availability**		
	**High**	**Variable**	**Low**
1. Premature mortality from noncommunicable diseases (mortality occurring at an age less than life expectancy)	✓		
2. Disability-free life expectancy at age 60 (healthy life expectancy)	✓		
3. Prevalence of disability in people ≥60 years	✓		
4. Prevalence of obesity in people ≥60 years	✓		
5. Influenza vaccine coverage	✓		
6. Proportion of out-of-pocket spending on health for people >60 years with respect to total spending on health (rate of out-of-pocket health spending to total health spending; ratio of those ≥60 to those ≤59 years)	✓		
7. Population coverage by health financing scheme for people ≥60 years	✓		
8. Percentage of older adults receiving a noncontributory pension	✓		
9. Percentage of older adults living in poverty	✓		
10. Average years of schooling of older adults	✓		
11. Proportion of older people who have had a functional evaluation in the past year			✓
12. Mortality rate attributable to poor-quality health care (total and people aged ≥60 years)		✓	
13. Mortality rate due to falls		✓	
14. Disability prevalence (rates and prevalence of disability and dependency)		✓	
15. Insufficient physical activity in adults ≥60 years			✓
16. Proportion of older people who have had a periodic health assessment		✓	
17. Polypharmacy and multimorbidity index (percentage of health care units with tools to reduce inappropriate polypharmacy)		✓	
18. Percentage of the population >60 years experiencing catastrophic out-of-pocket health expenses		✓	
19. Percentage or number of undergraduate medical and nursing programs that include geriatrics		✓	
20. Suicide rate in people ≥60 years		✓	
21. Effective coverage of cataract surgery for adults ≥50 years		✓	
22. Number of geriatricians per older adult and other health and social care professionals with training in aging or gerontology		✓	
23. Older adults’ self-reported level of satisfaction with health services		✓	
24. Percentage of older adults living alone		✓	
25. Cost associated with long-term care		✓	

***Source:*** Table prepared by the authors as a result of the meetings with experts. Technical data sheets for these indicators are available in Spanish from the authors upon request.

In terms of strengthening multisectoral coordination of long-term care, it was found that all four countries did not have systems for long-term care despite having older people with higher levels of dependency or difficulty in performing activities of daily living. In Barbados, 28% of severely disabled older people are offered long-term care services, but the prevalence of severe disability in these individuals was projected to reach 7.53% in 2020. In Mexico 14.3% of the population aged 50 and older reported having some difficulty with activities of daily living, and 40.8% of those received care from family members. In Chile, 93% of informal caregivers do not have a paying job, and in Brazil, 90% of informal caregivers are unpaid and receive no financial aid. Additionally, 24% of informal caregivers in Brazil stopped paid work to care for an older person. The lack of a long-term care system imposes an annual cost of US$ 123.9 million to the Brazilian health system as a result of prolonged hospitalizations; in Chile this cost is calculated to be US$ 81 million annually.

## DISCUSSION

Our team has developed a set of indicators to measure the responsiveness of a health system to the needs of older people. These indicators have been rated by experts as being useful to inform public policy and highly available in national data repositories.

The indicators aligned with the UHF are consistent with the health system responsiveness framework proposed by Mirzoev and Kane (4) and also with health system responsiveness as described by Khan and colleagues (13). More specifically, these frameworks provide quantitative indicators to understand how the health system dimensions of health financing, clinical services and human resources have implications for health outcomes and inequities among older populations. Health system responsiveness can be comprehensively assessed by (i) approaching intrinsic features of systems in terms of their inputs, outputs, and outcomes, and (ii) measuring the impact of the health system on the needs of older people in terms of their health, financial protection, and expectations.

The pilot national case studies analysis in Barbados, Brazil, Chile and Mexico identified key gaps. For example, working with highly specialized indicators (e.g. older people engaging in insufficient physical activity, recovery time after hip surgery) might impose technical difficulties at the beginning because these data are not readily available, but they could be easily computed from other routine indicators to validate them and maximize their information potential. For some indicators, the issues are conceptual (i.e. disability can be measured in multiple ways) or methodological (i.e. level of hospital or care service) in nature.

To avoid overburdening people who collect and manage data, those indicators that are collected or reported periodically should be preferred. The reasons for collecting data on indicators of public health have been to improve health coverage and access, and to improve health status and well-being. Therefore, the use of tracer indicators of health status is crucially important for monitoring universal health (5). Such tracers will provide information about critical processes that are representative of certain domains of the monitoring framework. For example, data about the effective coverage of cataract surgery can provide information not only about the dimension of the problem but also about what proportion of it is being resolved. One of the current limitations to achieving an effective-coverage approach is that indicators gathered from national surveys of healthy aging are predominantly based on classical notions of prevalence or incidence of diseases and the coverage of interventions rather than on well-being (i.e. functional status), effective coverage (i.e. recovering functionality after interventions) and unsatisfied needs. Collecting and analyzing more of these tracers could accelerate health system responsiveness towards enhancing healthy aging. Taking the pulse of health system responsiveness can provide managers and policymakers with information to efficiently drive a health system’s adaptation to population aging (4, 13). In the pursuit of healthy aging and a more person-centered health system, it is essential to measure the responsiveness of the system because it links the intrinsic features of the system with the needs of older people.

Our study has several strengths, including being the first integrative approach taken to measure health system responsiveness in the context of older people and population aging. Our integrative approach to responsiveness is innovative because it transcends the limits of user satisfaction, highlighted in earlier literature (17). Another asset is the inclusion of participants from diverse geographical, professional, and cultural backgrounds, and the verification of the feasibility of collecting and analyzing indicators (via the case studies). However, we faced limitations, such as the lack of systematic collection by the selected countries of data for all of the indicators.

The global goal of our project is to develop a quantitative tool for evaluating outcomes from diagnosis to treatment, fed by readily available data that can measure health system responsiveness and is directly linked to actions that can be taken towards developing a more age-friendly health system. Such evaluations would enable the identification of strengths, bottlenecks, and opportunities to establish cost-effective actions in priority areas for the care of older people.

Yet further consensus is needed to select a list of core indicators or tracers and to set thresholds for each indicator. A succinct consensus-based list of core indicators would help to advocate for the systematic collection and analysis of essential tracers of a health system and how it is responding to the needs of older people without increasing the monitoring burden for the system. Establishing thresholds for the indicators would facilitate their integration within a standardized tool and communication of the results to health system managers and policymakers. We plan to conduct a Delphi study in the quest of developing consensus.

## Conclusions

There is a need for a comprehensive framework to assess how and to what extent health systems are responding to the needs of older people. We think that such an evaluation is feasible in the Americas, even if countries have different levels of data availability. Developing consensus on key tracer indicators is needed to better target the needs of older populations in accordance with the local realities of each health system. The results of integrative responsiveness assessments would be helpful to increase health systems’ efficiency and inform public policy to guide adaptations of health systems to an aging world.

## Disclaimer.

Authors hold sole responsibility for the views expressed in the manuscript, which may not necessarily reflect the opinion or policy of the *Revista Panamericana de Salud Pública/Pan American Journal of Public Health* or the Pan American Health Organization (PAHO).
